# Gastrin Vaccine Alone and in Combination With an Immune Checkpoint Antibody Inhibits Growth and Metastases of Gastric Cancer

**DOI:** 10.3389/fonc.2021.788875

**Published:** 2021-12-01

**Authors:** Jill P. Smith, Hong Cao, Wenqiang Chen, Kanwal Mahmood, Teresa Phillips, Lynda Sutton, Allen Cato

**Affiliations:** ^1^ Department of Medicine, Georgetown University, Washington, DC, United States; ^2^ Cancer Advances, Inc., Durham, NC, United States

**Keywords:** gastric cancer, immune checkpoint, tumor microenvironment, metastases, gastrin, fibrosis, PAS, G17DT

## Abstract

Gastric cancer is a leading cause of cancer-related deaths worldwide. Recently, clinical studies have demonstrated that many of those with advanced gastric cancer are responsive to immune checkpoint antibody therapy, although the median survival even with these new agents is less than 12 months for advanced disease. The gastrointestinal peptide gastrin has been shown to stimulate growth of gastric cancer in a paracrine and autocrine fashion through the cholecystokinin-B receptor (CCK-BR), a receptor that is expressed in at least 56.6% of human gastric cancers. In the current investigation, we studied the role of the gastrin-CCK-BR pathway *in vitro* and *in vivo* as well as the expression of the CCK-BR in a human gastric cancer tissue array. CCK-BR and PD-L1 receptor expression and gastrin peptide was found in two murine gastric cancer cells (NCC-S1 and YTN-16) by qRT-PCR and immunocytochemistry. Treatment of NCC-S1 cells with gastrin resulted in increased growth. *In vivo*, the effects of a cancer vaccine that targets gastrin peptide (polyclonal antibody stimulator—PAS) alone or in combination with a Programed Death-1 antibody (PD-1 Ab) was evaluated in immune competent mice (N = 40) bearing YTN-16 gastric tumors. Mice were treated with PBS, PD-1 Ab (50 µg), PAS (250 µg), or the combination of PD-1 Ab with PAS. Tumor growth was significantly slower than controls in PAS-treated mice, and tumor growth was decreased even more in combination-treated mice. There were no metastases in any of the mice treated with PAS either alone or in combination with PD-1 Ab. Tumor proliferation by the Ki67 staining was significantly decreased in mice treated with PAS monotherapy or the combination therapy. PAS monotherapy or combined with PD-1 Ab increased tumor CD8+ T-lymphocytes and decreased the number of immunosuppressive M2-polarized tumor-associated macrophages. CCK-BR expression was identified in samples from a human tissue array by immunohistochemistry confirming the clinical relevance of this study. These results confirm the significance of the gastrin-CCK-BR signaling pathway in gastric cancer and suggest that the addition of a gastrin vaccine, PAS, to therapy with an immune checkpoint antibody may decrease growth and metastases of gastric cancer by altering the tumor microenvironment.

## Introduction

Gastric adenocarcinoma (gastric cancer) is a common malignancy and is the world’s second leading cause of cancer mortality worldwide (1). Novel therapeutic targets are desperately needed because the meager improvement in the cure rate of about 10% realized by adjunctive treatments to surgery is unacceptable as >50% patients with localized gastric cancer succumb to their disease (2). The prognosis of those with advanced gastric cancer is poor with a five-year survival of only 20–30% (3, 4). The current standard of care for advanced gastric cancer in the first line setting remains a combination of a fluoropyrimidine (e.g., 5-fluorouracil; 5FU) and a platinum (e.g., cis-platinum) containing chemotherapeutic agent. Targeted therapy may offer new possibilities for the treatment of gastric cancer. Since HER2 receptors are found in approximately 20% of gastric cancers, the addition of a HER2 receptor antibody to standard chemotherapy may be beneficial as demonstrated in the ToGA study where Trastuzumab (Herceptin) was beneficial in subjects with HER2-positive gastric cancer (5). The Cancer Genome Atlas (TCGA) Research Network described four groups of gastric cancer based upon molecular classifications including: EBV (Epstein–Barr virus), MSI (microsatellite instability), GS (genomically stable), and CIN (chromosomal instability) (6). The immune response to the tumor could play an important role within the EBV and MSI subgroups (7). With the recent use of immune checkpoint antibodies, investigators have been exploring whether this immunotherapy would be beneficial for gastric cancer (7). The KEYNOTE-012 study tested 39 subjects in a Phase 1 trial that were PD-L1 positive with pembrolizumab and found an overall survival of 11.4 months (8). The KEYNOTE-059 trial showed that pembrolizumab monotherapy was effective treating those with previously treated gastric or gastroesophageal cancer (9). Another PD-1 antibody, nivolumab, has been approved for first line therapy in gastric cancer in combination with chemotherapy after the results of the CheckMate-649 clinical trial (10). A number of clinical trials have been conducted now with various immune checkpoint antibodies (11) and although these agents have provided additional therapeutic options for those with gastric cancer, unfortunately the median overall survival still remains less than 12 months (12). For these reasons novel strategies are needed to improve response of those with gastric cancer to immunotherapy. One possible reason for the still low response to immune checkpoint antibodies may be related to the paucity of tumor infiltrating CD8+ lymphocytes in the tumor (13). Another possible reason for the low response rate may be due to the fibrosis of the tumor microenvironment that prevents penetration of therapies and immune cells (14). Therapeutic agents that target cancer cell receptors such as HER2 (5) have been shown to improve survival and yet most chemotherapy agents used in gastric cancer are not target-specific.

**Figure 1:**
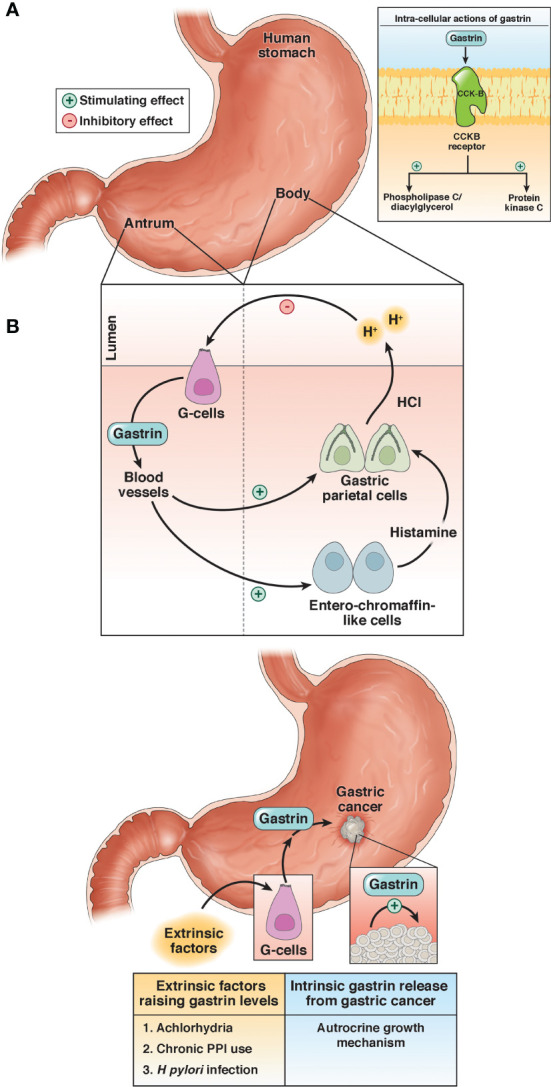
Physiologic and pathologic roles of gastrin. **(A)** Under physiologic conditions, gastrin is released from the G-cells in the antrum of the stomach and stimulates the release of acid (HCl) by activation of the enterochromaffin-like cells and gastric parietal cells. **(B)** Gastrin stimulates growth of gastric cancer by activating the CCK-BR by an autocrine mechanism or exogenous stimulation by high gastrin blood levels induced by achlorhydria, chronic use of proton pump inhibitors (PPI), or infection with *Helicobacter pylori (H. pylori)*. Reproduced with permission from the Cell Mol Gastroenterol Hepatol (15).

The gastrointestinal (GI) peptide gastrin is responsible for gastric acid secretion and growth of the GI tract, and gastrin mediates its effects through the cholecystokinin-B receptor or CCK-BR (**Figure 1**) (16). Unlike the physiologic expression of gastrin in the G cells of the stomach antrum (17), the gastrin gene also becomes overexpressed *de novo* in non-endocrine epithelial cells of gastric cancer (18) where it can stimulate growth in an autocrine fashion. Likewise, the CCK-BR also becomes over-expressed in cancer cells (19) and this receptor is responsive to both paracrine and autocrine stimulation by gastrin. Investigators have studied the expression of gastrin and the CCK-BR from resected human gastric cancers and found that most expressed CCK-BRs and gastrin (20–22). Gastrin may also stimulate growth of gastric cancer when blood gastrin levels are increased from chronic use of high dose proton pump inhibits (PPIs), achlorhydria or *Helicobacter pylori* infection (**Figure 1**) (15). Since gastrin has been shown to stimulate growth of human gastric cancer (19), researchers have been studying means to block gastrin’s actions in gastric cancer using CCK-BR antagonists (23, 24) and their use in human trials reviewed (25–27).

Polyclonal Antibody Stimulator (PAS) is a therapeutic immunogen cancer vaccine comprised of a nine amino acid epitope derived from the amino-terminal sequence of gastrin-17 that is conjugated to diphtheria toxoid. PAS exerts an immunomodulatory effect by activating both B (28–30) and T cells (31). PAS stimulates the production of antibodies to different epitopes of the G17 and precursor G17-Gly gastrin peptides. These antibodies can bind to gastrin peptides to prevent their interaction with the CCK-BRs on the surface of tumor cells. Preclinical studies were performed in several animal models that have CCK-BRs including gastric cancer (32). In animal models, PAS-generated anti-gastrin antibodies have been shown to reduce the growth and metastases (28, 29, 33). Passive immunization with PAS antibodies raised in rabbits improved survival of SCID mice bearing gastric cancers compared to diluent treated controls (32).

To date 22 clinical studies have been conducted with PAS. Of these, 840 patients have been enrolled in five clinical trials for the treatment of pancreatic cancer; 234 subjects enrolled in five clinic studies in gastric cancer (34, 35); and 475 subjects enrolled in 10 clinical studies with advanced colon cancer. In the gastric cancer clinical trial (designated GC4 Study), the median survival of those with advanced gastric cancer treated with PAS plus cisplatin and 5FU was significantly prolonged (10.8 months) in subjects that mounted a protective antibody titer against gastrin compared to subjects treated with PAS plus cisplatin and 5FU that failed to generate an antibody response (4.8 months). The only notable PAS-related adverse events in all 22 studies were injection-site reaction and pyrexia. The purpose of the current study is to evaluate the effect of PAS and PD-1 antibody therapy alone or in combination in a murine model of gastric cancer.

## Materials and Methods

### Cell Lines

Two murine cell lines were evaluated in this investigation. Murine gastric cancer cell line NCC-S1 (NCC) (36) was provided by Dr. Kim through his collaborator Dr. Timothy Wang of Columbia University, NY. The second gastric cancer cell line YTN-16 (YTN), was established and provided by Professor Sachiyo Nomura (37) through her collaborator Dr. James R. Goldenring of Vanderbilt University School of Medicine, TN. The YTN cells are known to be invasive and metastatic even after subcutaneous injection in mice. Before the cells were used in animals, they were tested by IMPACT II PCR Profile and were negative for all pathogens. YTN cells were grown in culture using DMEM and NCC cells were grown in RPMI media; both with 10% fetal bovine serum in a humidified 5% CO2 incubator at 37**°**C.

### CCK-BR and PD-L1 Receptor Characterization in Gastric Cancer Cells by Quantitative PCR

Total RNA was extracted from cells (Qiagen) and subjected to quantitative PCR (qRT-PCR) in the fast cycling mode using a thermal cycler (Applied Biosystems) to examine the expression of the CCK-BR and PD-L1 expression. Primers used included: CCK-BR: F-5’GATGGCTGCTACGT-GCAACT-3’and R-5’CGCACCACCCGCTTCTTAG-3’; and PD-L1: F-5’TGCGGACTACAAGCG-AATCACG-3’ and R-5’-CTCAGCTTCTGGATAACCC-TCG-3’. PCR was performed with 40 cycles and an annealing temperature at 60°C. HPRT was used as a normalizer control gene. Control RNA was extracted from normal mouse liver because it does not express either CCK-BR or PD-L1. Each reaction was performed in triplicate and each PCR test was performed three times for each receptor.

### Effects of Gastrin Administration on *In Vitro* Growth of Gastric Cancer Cells

In order to determine if exogenous administration of gastrin could stimulate growth of gastric cancer cells, murine NCC cells (10,000) were plated into each well of a 96-well plate. After an overnight incubation, wells were exposed to gastrin 10 nM (N = 12 each) or media alone (control, N = 12). After an additional 24 h, the growth of the cells was evaluated with the MTT (3-[4,5-dimethylthiazol-2-yl]-2,5-diphenyltetrazolium bromide; thiazolyl blue) cell proliferation assay and differences analyzed by a colorimetric assay in a plate reader at 450 nm.

### Gastrin Peptide Expression by Immunocytochemistry in Gastric Cancer Cells

NCC and YTN gastric cancer cells were plated onto glass coverslips in 4 cm^2^ petri dishes. When cells reached log-phase growth, the cells were fixed and reacted to a rabbit polyclonal antibody (Peninsula Laboratories, Belmont, CA; cat#: T4347) with a titer 1:50 overnight at 4°C, followed by incubation with one to three drops of Biotinylated Secondary Antibody (Vial A, Novus Biologicals; Centennial, CO) for 60 min. The slides were treated with one to three drops of HSS-HRP (Vial B, Novus) for 30 min, washed and DAB Chromogen was added for 3 min. Control cells were reacted with secondary antibody only. Images were taken of each sample from the slides using an Olympus BX61 microscope with a DP73 camera.

### *In Vivo* Animal Studies

All animal studies were done in an ethical fashion and under the approval of the IACUC from Georgetown University. Several attempts were made to establish tumors in C57BL/6 mice using the NCC cells unsuccessfully. The first attempt included the injection of luciferase tagged 5 × 10^5^ NCC cancer cells orthotopically into the stomach subserosa (N = 40). After imaging with luciferin and dissecting mice, no tumors were found. The NCC cells were then injected subcutaneously on the right flank with a total of 0.1 ml volume of 1.5 × 10^6^ NCC cancer cells, but after 33 days, no tumors formed. It appears that the NCC cells will only form tumors in SCID mice or *Villin*-*Cre*, *Smad4* ^F/F^, *Trp53F/F*, *Cdh1* ^F/wt^ mice according to Park et al. (36). The YTN gastric cancer cells are known to be invasive and metastatic even after subcutaneous injection in C57BL/6 mice (37). Therefore, YTN were used for the *in vivo* studies by injecting (5 × 10^6^) cells into each of 40 female C57BL/6 mice in 0.2 ml volume. Tumor growth was measured weekly with calipers and volume calculated by L × W^2^ × 0.5.

### Treatments

Mice bearing YTN tumors were divided into four treatment groups (N = 10 each). Control mice were treated with PBS in 0.1 ml ip injection given at the same time as the other treatments. PD-1 antibody 50 μg ip (PD-1 Ab; Clone RMPI-14 was purchased from Bio X Cell, West Lebanon, NH) was administered at baseline (one week after tumor inoculation; week 0) and at weeks 1, 3, and 6. PAS 250 μg was administered subcutaneously in 0.1 ml volume at the same time as PD-1 Ab and also at week 9. After 10 weeks of growth the control mice were appearing moribund so the mice were ethically euthanized, tumors removed and weighed and metastases counted.

### Tissue Analysis

All observed metastases counted were dissected and formalin fixed and paraffin embedded for confirmation by hematoxylin and eosin (H&E) staining. Tumors were reacted with Masson’s trichrome stain for analysis of fibrosis in the tumor microenvironment. To determine the proliferation index of the tumors, tissue sections (5 µm) were reacted with a rabbit monoclonal antibody for Ki67 (Biocare, cat# CRM325; 1:80). Immunohistochemical staining was also performed of tumor tissue sections (5 µm) to evaluate tumor infiltrating lymphocytes with CD8, (1:25, Cell Signaling, cat # 98941) and with rabbit polyclonal antibody against arginase-1 (ThermoFisher, cat # PA5-29645) at a dilution 1:1,800 to examine M2-polarized tumor associated macrophages.

### CCK-BR Expression in Human Gastric Cancer

A human tissue microarray containing 24 cases/72 cores of human gastric cancer and samples from normal stomach was obtained from US Biomax (Rockville, MD; Cat#BC01011). After antigen retrieval, the array was incubated with the primary goat polyclonal antibody CCK-BR (#Ab77077, Abcam) at 1:200 titer overnight at 4°C. After rinsing, the slide was incubated with one to three drops of Biotinylated Secondary Antibody (Vial A, Novus) for 60 min. The slide was then treated with one to three drops of HSS-HRP (Vial B, Novus) for 30 min, washed and DAB Chromogen was added for 3 min. Images were scanned using an Aperio GT450 machine and images captured with software from Aperio Image Scope. CCK-BR staining was analyzed by densitometry with Image-J software corrected for area of tissue examined.

### Statistical Analysis

Tumor growth rates were analyzed using linear regression analysis to compare slopes of the growth curves between each treatment group. Slides were scanned using an Aperio GT450 machine and images analyzed with software from Aperio Image Scope for the number of immunoreactive cells per high powered field (for Ki67 and CD8 cells). Images at the same magnification and identical surface area were taken (up to N = 10 per slide) for each tumor using the Aperio software. Slides for fibrosis and M2 polarized macrophage were quantitatively analyzed for integrated density with ImageJ computer software. Raw data results from images were analyzed using ANOVA and T-Test (with Bonferroni correction for multiple comparisons to controls) with GraphPad Prism version 9.

## Results

### Characterization of Gastric Cancer Cells *In Vitro*

**Figure 2:**
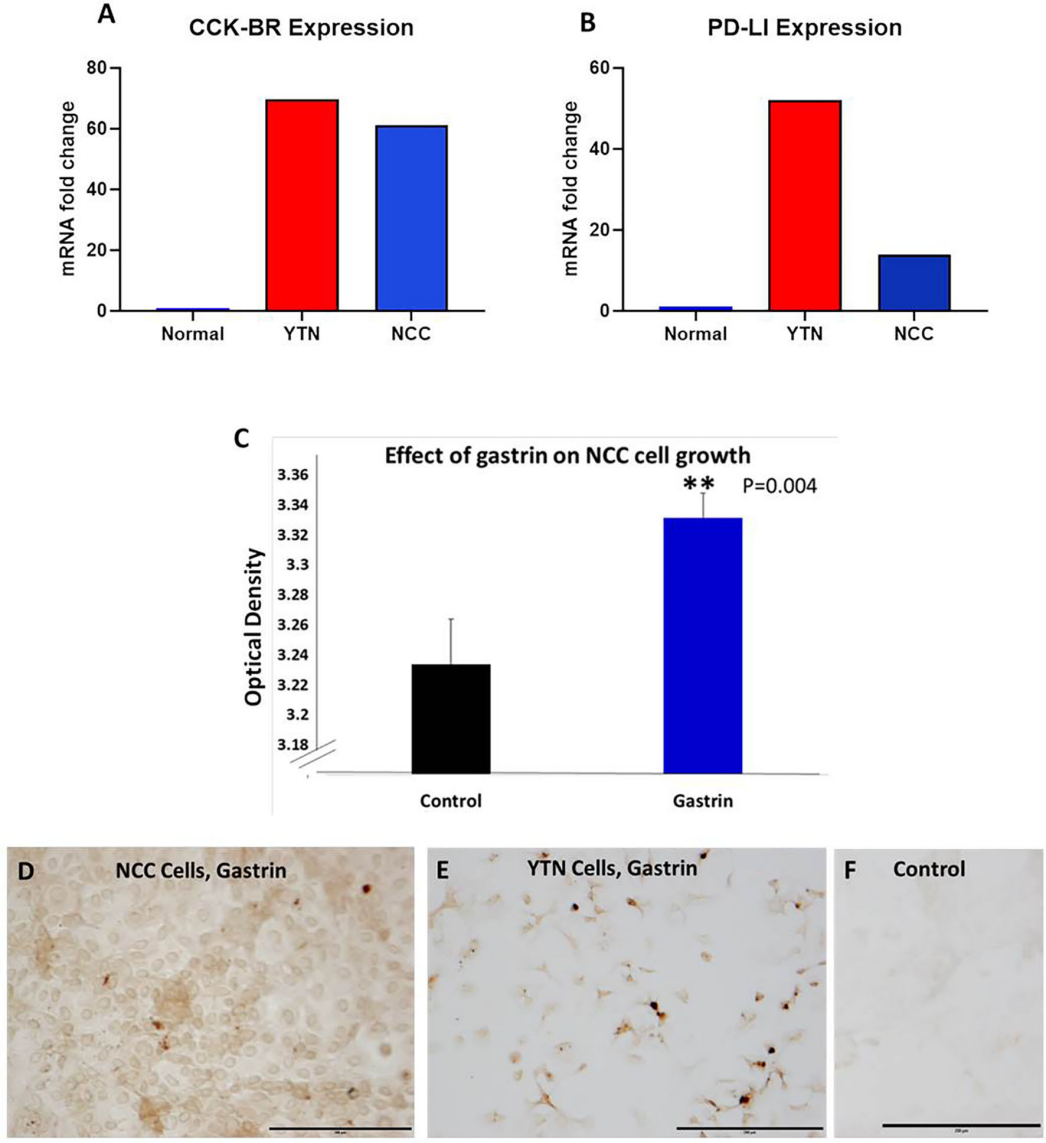
Characterization of murine gastric cancer cells *in vitro*. **(A)** mRNA expression of CCK-BR is increased greater than 60-fold in YTN and NCC gastric cancer cells compared to normal mouse tissue. **(B)** mRNA expression by qRT-PCR of PD-L1 is markedly increased in gastric cancer YTN and NCC cells compared to normal tissues. **(C)** Exogenous gastrin stimulates growth of murine NCC gastric cancer cells *in vitro* (P = 0.004). **Significantly different from control. **(D)** Gastrin peptide expression is detected in NCC gastric cancer cells. **(E)** Gastrin peptide expression is detected in YTN gastric cancer cells. **(F)** Control cells stained with the secondary antibody only show no evidence of nonspecific immunoreactivity. Scale bar 200 μm.

Two separate murine gastric cancer cells were evaluated for expression of CCK-BR, PD-L1 receptors and gastrin peptide *in vitro*. Gene expression of CCK-BR and PD-L1 were increased in both NCC and YTN gastric cancer cells compared to noncancerous mouse tissues (**Figure 2**). CCK-BR expression was increased greater than 60-fold in mouse YTN and NCC gastric cancer cells compared to normal mouse tissues (**Figure 2A**). PD-L1 mRNA expression was increased 52-fold in YTN cells and 24-fold in NCC cells over normal tissues (**Figure 2B**). Growth of NCC cells increased significantly (P = 0.004) when exposed to exogenous gastrin (**Figure 2C**). Immunocytochemistry revealed endogenous gastrin peptide expression in both NCC (**Figure 2D**) and YTN (**Figure 2E**) gastric cancer cells suggesting that these gastric cancer cells produce their own gastrin peptide to stimulate growth *via* the CCK-BR in an autocrine fashion. Control cells that reacted with the secondary antibody alone were negative for staining (**Figure 2F**).

### Effects of PAS and PD-1 on Growth and Metastases of YTN Tumors

**Figure 3:**
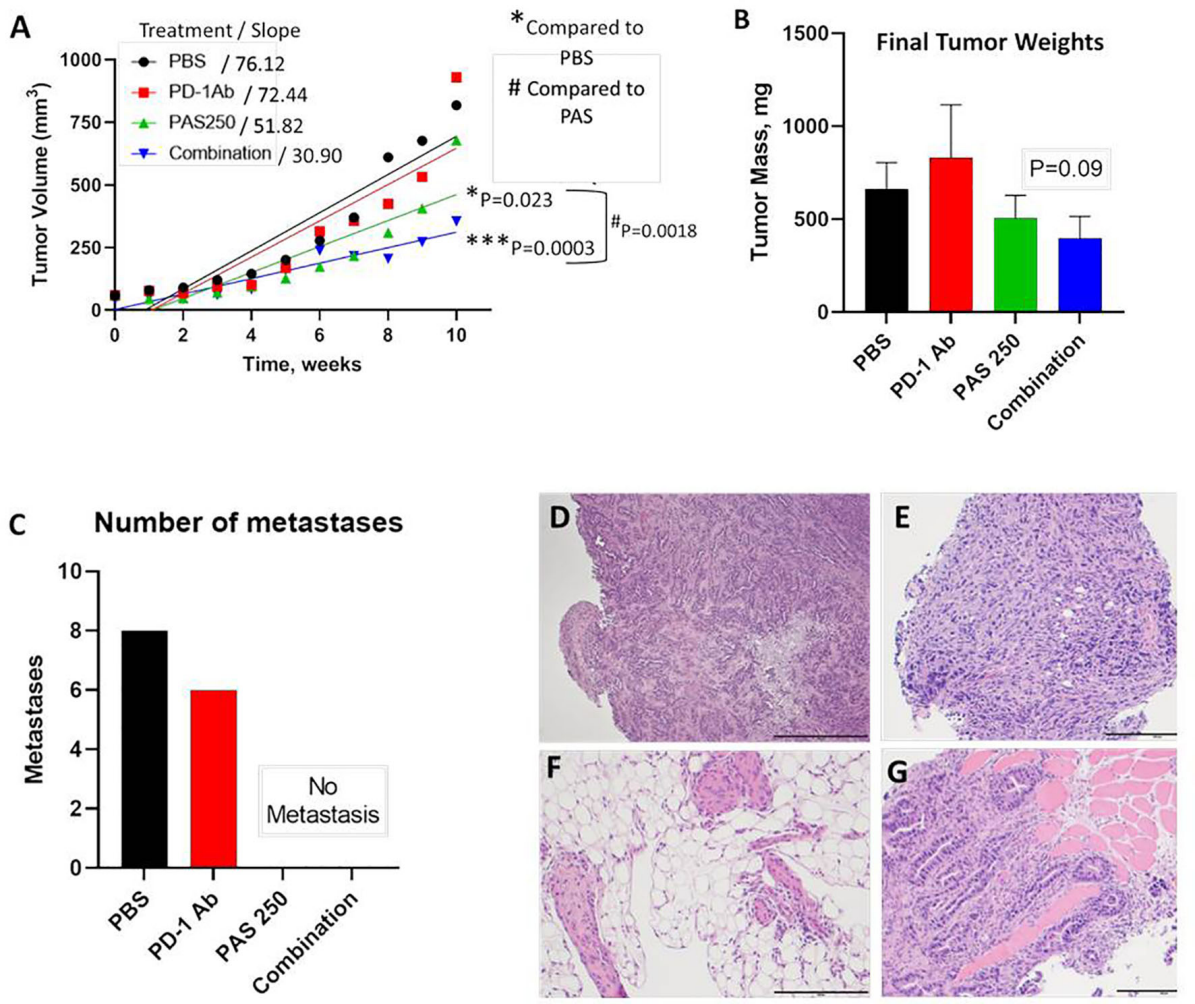
PAS vaccination alone or in combination with PD-1 Ab inhibits growth and metastases of YTN gastric cancer tumors in mice. **(A)** YTN tumor volumes over time for each treatment group and respective slope of the line are shown. PD-1 Ab monotherapy did not alter rate of YTN tumor growth compared to PBS-treated controls. Tumors of mice treated with PAS monotherapy (P = 0.023) or in combination with PD-1 Ab (P = 0.0003) significantly reduced tumor growth in mice compared to PBS control treated mice. Tumors of mice treated with both PAS and the PD-1 Ab exhibited significantly smaller tumors compared to PAS monotherapy (P = 0.0018). **(B)** Final tumor mass *ex vivo* showed a reduction in size in mice treated with PAS in combination with the PD-1 Ab (P = 0.09). **(C)** Number of metastases for each treatment group demonstrates that metastases were only observed in Control (PBS-treated) mice and in mice treated with PD-1 Ab. No metastases were found in mice treated with PAS monotherapy or PAS in combination with the PD-1 Ab. **(D–G)** Metastases were confirmed histologically by H&E stain. **(D)** Invasive YTN tumor invading the stomach wall. **(E)** Peritoneal seeding with metastases. **(F)** Invasion of YTN tumor cells in the mesentery fat. **(G)** YTN cancer invading the abdominal wall skeletal muscle.

YTN gastric cancer tumor volumes measured over time are shown in **Figure 3A**. Therapy with PD-1 Ab monotherapy had no effect on tumor growth compared to controls. In contrast, mice treated with PAS monotherapy or PAS in combination with PD-1 Ab had significantly slowed tumor growth over time. PAS monotherapy slowed tumor growth by 31% compared to PBS-treated controls (P = 0.023). When PAS was given in combination with the PD-1 Ab the tumor growth was slowed by 59% compared to tumors of PBS-treated controls (P = 0.0003). When the growth rate of tumors from PAS-vaccinated mice was compared to that of the tumors of mice treated with the combination therapy, the difference was statistically significant (P = 0.0018). These results would suggest that the combination therapy is better than PAS monotherapy. The mass of the tumors when excised was less in the PAS- and combination-treated mice, but this difference did not reach significance (**Figure 3B**). The total number of metastases in each group were counted at autopsy and confirmed by histology. **Figure 3C** shows the remarkable finding that there were no metastases in the mice treated with PAS monotherapy or PAS combined with the PD-1 Ab. Hematoxylin & eosin staining confirmed that the tissues dissected from control mice and PD-1 Ab treated mice were metastases. **Figures 3D–G** show representative histology of YTN metastases from the stomach wall, mesentery, peritoneum, and abdominal wall, respectively.

**Figure 4:**
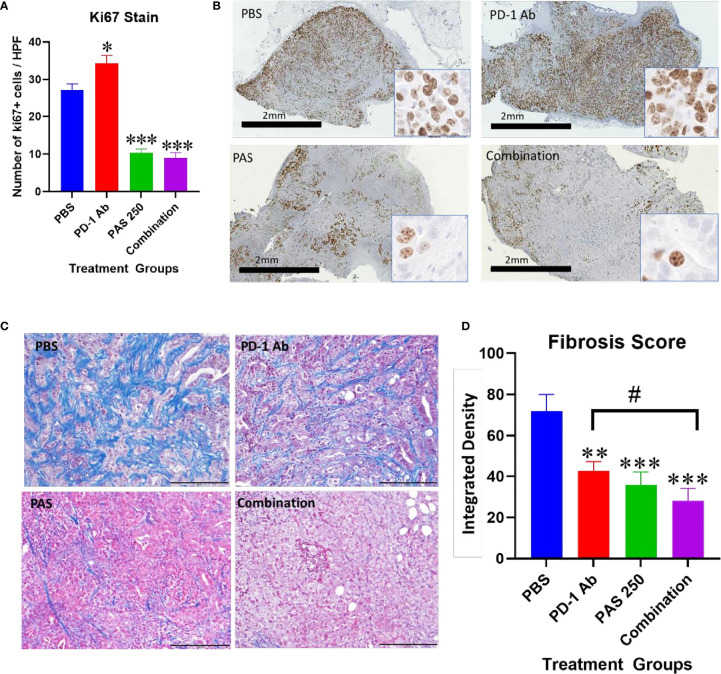
Effects of PAS and PD-1 Ab treatment on tumor proliferation and fibrosis. **(A)** The mean number ± SEM of Ki67 stained cells is shown for each cohort of YTN tumors. Ki67 immunoreactivity in PD-1 Ab tumors increased compared to PBS-treated controls (P <0.05). * Significantly different from control. Ki67 staining was significantly reduced in tumors of mice treated with PAS monotherapy or in combination with the PD-1 Ab (P <0.0001). **(B)** Representative images from tumors reacted with Ki67 antibody for each treatment group is show at low magnification (2×, bar scale, 2 mm) and at a higher magnification (40×, Box insert). **(C)** Representative images of tumors from each treatment group stained for fibrosis with Masson’s trichrome stain (scale bar = 200 μm). **(D)** Mean values ± SEM for fibrosis staining is shown for each treatment as analyzed by integrated density. Intratumoral fibrosis was decreased in all treatment groups compared to PBS-treated control tumors. Tumors of the combination therapy group also exhibited less fibrosis than tumors of the mice treated with PD-1 Ab monotherapy. (Compared to PBS **P < 0.01; ***P < 0.001; compared to PD-1 Ab ^#^<0.05).

Another demonstration of the effects on tumor growth is the measurement of the Ki67 proliferation index. Ki67 immunoreactivity is increased in the tumors of PBS and PD-1 Ab treated mice (**Figure 4A**). The proliferation index is significantly decreased in tumors of mice treated with PAS monotherapy or in combination with PD-1 Ab (**Figure 4A**). A low power (magnification 2×) representative image from each treatment group is shown in **Figure 4B** with a higher magnification (40×) insert image for each tumor. Marked Ki67 immunoreactivity is identified in tumors from PBS and PD-1 Ab treated mice. In contrast, the Ki67 staining is markedly decreased in tumors of mice treated with PAS with or without PD-1 Ab. These histologic sections confirm tumors of the PAS and combination-treated mice had decreased proliferation or growth rate.

### PAS and PD-1 Ab Therapy Decrease Fibrosis in the Gastric Cancer

Tumor fibrosis is thought to impede the penetration of chemotherapeutic agents into cancers and also restrict the influx of T-lymphocytes. YTN gastric tumors demonstrate characteristic dense fibrosis as seen in tumors of PBS-treated control mice with the Masson’s trichrome stain of **Figure 4C**. There is visibly less fibrosis noted in the tumors of mice treated with PAS monotherapy or PAS in combination with PD-1 Ab. Computerized analysis and quantification of the integrated density of fibrosis is shown for each treatment group in **Figure 4D**. Although there was modest decrease in fibrosis in tumors of PD-1 Ab treated mice, when combined with PAS therapy, the amount of fibrosis was significantly further decreased.

### PAS and PD-1 Ab Therapy Change the Immune Cell Signature of Gastric Cancer

**Figure 5:**
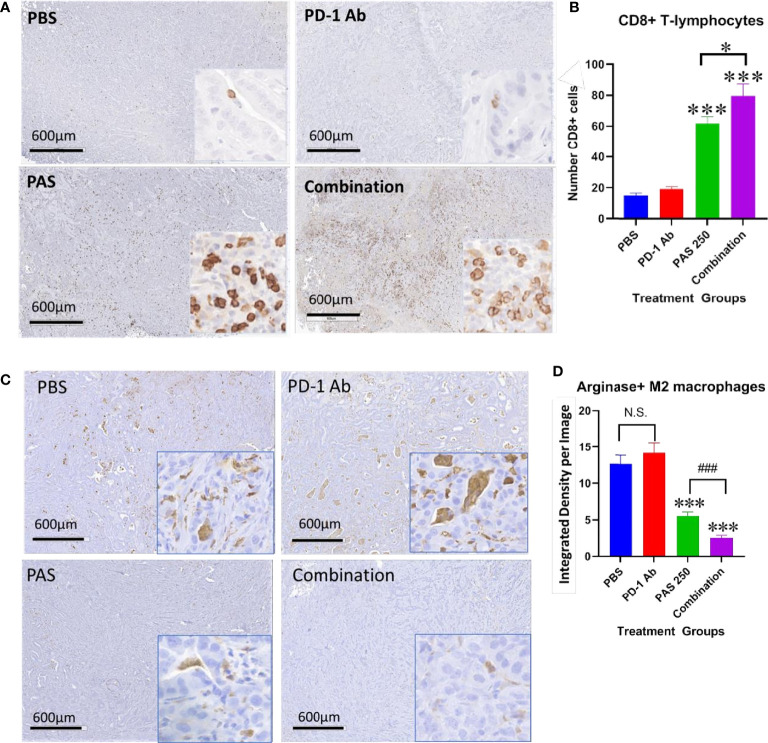
PAS monotherapy and in combination with PD-1 Ab alter the tumor immune cell signature. **(A)** Representative low magnification tumor from each treatment or control group (scale bar 600µm) and a higher magnification (20×; Box insert) of tumors stained with an antibody for CD8+ T-lymphocytes. **(B)** Columns represent the mean ± SEM of the number of CD8+ immunoreactive cells in sections of YTN tumors from each group. PAS monotherapy and in combination with a PD-1 Ab significantly increase the number of CD8+ immunoreactive T-cells in the YTN tumors compared to tumors of PBS-treated mice. The combination of PAS with PD-1 Ab also markedly increased the number of CD8+ cells compared to PAS monotherapy. (***P <0.001 compared to PBS; ^###^P <0.001, compared to PAS). **(C)** Representative low magnification tumor from each treatment or control group (scale bar 600µm) and a higher magnification (20X; Box insert) of tumors stained with and antibody for M2-polarized tumor-associated macrophages (TAMs). **(D)** Columns represent the mean ± SEM of integrated density from ImageJ analysis for concentration of M2-polarized TAMs. The number of TAMs decreased significantly in tumors of mice treated with PAS monotherapy or in combination with the PD-1 Ab. Analysis showed that the combination therapy reduced TAMs significantly more than PAS alone. (***P<0.001 compared to PBS; ### P<0.001, compared to PAS). NS, not significant.

One reason for the lack of effect of immune checkpoint therapy in cancers is thought to be due to the paucity of tumor infiltrating T-cells. Tumors from each treatment group were stained for CD8+ T-lymphocytes and the number of immunoreactive cells compared between groups. **Figure 5A** shows the lack of CD8+ T cells in gastric tumors of PBS control mice and in PD-1 Ab-treated mice. The number of CD8+ immunoreactive cells is visibly increased in tumors of PAS-treated mice and mice treated with the combination therapy (**Figure 5A**). Computer analysis of the YTN tumors stained with the CD8+ antibody show marked increase in CD8+ T-lymphocytes in tumors of PAS-treated mice and even a significantly greater increase of CD8+ T cells in mice treated with the combination therapy (**Figure 5B**).

Tumors from each group also underwent immunohistochemical staining with an antibody for arginase to detect M2-polarized tumor-associated macrophages (TAMs). These immunosuppressive TAMs are abundant in the tumors of control mice and PD-1 Ab-treated mice (**Figure 5C**). In contrast, there are noticeably fewer arginase+ TAMS in the gastric tumors of mice treated with PAS which confirms that the immunoreactivity is significantly decreased in tumors of PAS-treated mice. Tumors of mice treated with both PAS and the PD-1 Ab have even further decreased immunoreactivity of arginase positive TAMs (**Figure 5D**).

### Human Gastric Cancer Expresses CCK-BR by Immunohistochemistry

**Figure 6:**
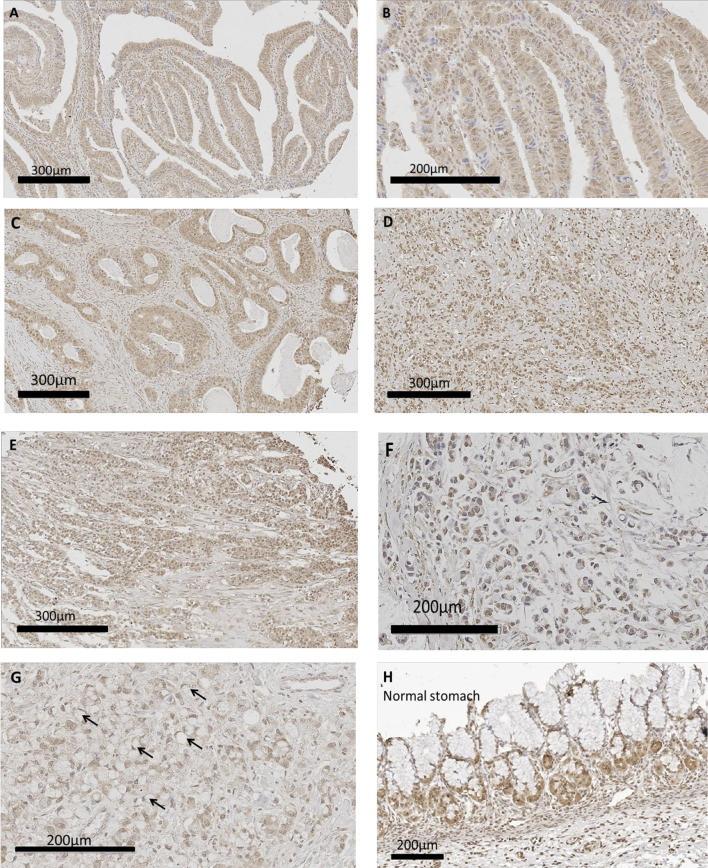
CCK-BR protein expression by immunohistochemistry in human gastric cancer and normal tissues from a human gastric tissue array (US Biomax # BC01011). The array was stained with a CCK-BR antibody (Abcam 77077) at a titer of 1:200 overnight at 4°C. **(A–C)** Gastric cancer images representative of the intestinal type histology are shown. **(D, E)** Representative images of gastric cancers with the diffuse histologic type are shown. **(F)** Gastric carcinoma mucinous adenocarcinoma. **(G)** Gastric cancer signet ring histology; arrows point to signet ring cells. **(H)** Histology normal human stomach.

Human gastric cancer epithelial cells were positive for CCK-BR immunoreactivity (**Figure 6**) implying that the administration of PAS to human subjects would also decrease activation of this receptor by neutralizing gastrin. The most common histologic classification was described by Lauren (38) where cancers were categorized histologically into one of two types: intestinal or diffuse. **Figures 6A–C** show CCK-BR immunoreactivity in tissues from the human gastric cancer array with the intestinal-type histology showing the characteristic glands or tubules lines by epithelial cells. Histological diffuse gastric carcinoma cells lack cohesion and invade tissues independently or in small clusters (39). Representative diffuse type gastric cancers also expressed CCK-BR expression and are shown in **Figures 6D–E**. Mucinous gastric cancer (**Figure 6F**) and signet ring gastric cancer (**Figure 6G**) are less common histologic types of gastric cancer. Characteristic staining of CCK-BR positive cells in the glands of the normal human stomach are seen in **Figure 6H**. There was not significant difference in the intensity of the CCK-BR staining according to the integrated density analyzed with ImageJ between tumors classified as Grade 1 (164.8 ± 1.4), Grade 2 (159.7 ± 1.4), and Grade 3 (162.8 ± 1.1).

## Discussion

In the current investigation, we demonstrated using two murine gastric cancer cell lines and a human tissue microarray that the gastrin: CCK-BR signaling pathway is important in stimulating growth of gastric cancer. CCK-BRs were expressed in both cell lines and exogenous gastrin stimulated cell growth *in vitro* confirming gastrin sensitivity. Immunocytochemistry revealed endogenous gastrin expression within the gastric cancer cells suggesting that gastric cancer may regulate its own growth by an autocrine mechanism. Since exogenously administered gastrin or endogenously produced gastrin from the cancer cells can activate the CCK-BR receptor resulting in cellular or tumor proliferation, strategies to interrupt the interaction of gastrin should inhibit growth. Indeed, we showed that a vaccine that targets gastrin can inhibit growth of gastric cancer in mice and prevent metastases. The PAS vaccine when administered as monotherapy decreased tumor growth in mice; however, the tumor inhibitory effect was significantly affected by co-administration of the PD-1 Ab with PAS. The advantage of having a therapy such as the PAS vaccine that shows efficacy with monotherapy is that when treating subjects with gastric cancer, not all subjects are eligible for immune checkpoint antibody treatment or some may have experienced adverse effects from the immune checkpoint therapy; hence, monotherapy may provide an alternative option to treat these subjects. However, in those subjects eligible for immune checkpoint therapy, the addition of PAS could significantly decrease tumor growth and prevent metastases. This vaccine, PAS, significantly decreased gastric cancer proliferation and this change was confirmed histologically with marked decreased in the number of Ki67 immunoreactive tumor cells. PAS therapy also decreased fibrosis in the tumor microenvironment. Vaccination with PAS also altered the tumor immune cell signature by increasing the number of CD8+ T-cells and decreasing the number of M2-polarized immunosuppressive macrophages rendering the tumor microenvironment more susceptible to other treatments, such as PD-1 Ab therapy.

Although the cancer cells expressed receptors for PD-L1, monotherapy with a PD-1 Ab did not significantly decrease gastric cancer growth or metastases. However, when PD-1 Ab therapy was administered in combination with PAS, there was a greater inhibitory effect on tumor growth rate than with PAS therapy alone. One explanation for the additive effect of PAS with the PD-1 Ab may be attributed to the marked increase in CD8+ T-cells when the two immune therapies are given together. Another beneficial finding of combined administration included the additive effect seen on the number of arginase positive M2-polarized macrophages. We previously described an additive effect on tumor inhibition in pancreatic cancer when PD-1 Ab therapy alone had no inhibitory effects but when combined with PAS, the combination therapy had a greater effect than PAS alone (31). In the prior study in pancreatic tumors, we also showed that PAS in combination with the PD-1 Ab decreased fibrosis in the tumor microenvironment. The decrease in fibrosis may perhaps allow for the influx of T cells.

Although this investigation was performed in immune competent mice with syngeneic murine tumors, the results of the CCK-BR immunoreactivity on the human gastric cancer array support the important translational and clinical relevance of this work. We found that both murine gastric cancers (YTN and NCC) expressed CCK-BRs and when YTN tumor bearing mice were treated with a gastrin vaccine, the tumor growth rate and metastases significantly decreased. Gastrin is the major ligand activating the CCK-BR and because PAS therapy induces neutralizing gastrin antibodies and gastrin-activated memory T cells (31), the ability to decrease signaling at this receptor is central to inhibiting cancer growth. Sheng et al. (40) demonstrated that that mature enterochromaffin-like cells (ECL) cells in the gastric corpus express CCK-BRs, and that that gastric isthmal progenitor cells also expressed CCK-BRs that responded to hypergastrinemia by supplying new ECL cells. Their elegant work supports the importance of gastrin as a trophic peptide activating the CCK-BR in the gastric mucosa. We previously showed that CCK-BRs are expressed on several human gastric cancer cell lines (19) and that gastrin-stimulated growth *in vitro* was only blocked by the selective CCK-BR antagonist, L265,260. The human gastric cancer tissue array immunoreactivity for the CCK-BR in numerous human gastric cancers in this current study suggests the importance of this receptor as a potential target for therapy in human subjects. The finding of CCK-BR staining in both the intestinal and diffuse histologic gastric cancer types suggests the broad implication of utilizing a therapy that targets this proliferative pathway. Although mucinous and signet ring histologic types occur less often, the prognosis with these histologic types is typically more severe (41). Tissues in the human gastric cancer array with these less frequent histologic types also stained positive for the CCK-BR suggesting the potential broad application of PAS therapy in gastric cancer.

Research on PAS was initiated by Dr. Susan Watson in the early 1990s (29, 32, 33). Although not popular at the time, Dr. Watson decided to take an immune approach to treating GI cancers by producing high-affinity anti-G17 antibodies that could neutralize serum gastrin and cell-associated gastrin. Since it had previously been reported that serum gastrin levels are elevated in colorectal tumors (42), she decided to begin her investigation in that tumor (28, 29). A wealth of clinical data was generated over the years that have been used to determine an appropriate adjuvant, dosing schedule, dose concentration and boosters required to produce high affinity anti-G17 antibodies. It was discovered that as antibody titers rose, serum gastrin levels decreased (43). It was found that antibody titers could be followed and subjects with titers 1.2 units above baseline, on average, doubled their survival times in colon, pancreatic and gastric cancers. There were some surprising results along the way. PAS was synergistic with Gemcitabine, and unexpected long-term survivors were observed in several studies including in pancreatic cancer. These results led to discussions that perhaps something beyond neutralizing gastrin was occurring; however, the tools available today were not available then. Although there were positive studies in all three GI cancer indications and a well characterized safety profile of the product, the development of PAS took a major set-back when the company funding its development failed. The last subjects treated with PAS were in 2004.

In the last two years, a great deal has been learned about the mechanism of action of PAS. Not only does it produce high affinity anti-G17 antibodies, but PAS activates a cellular immune response that increases memory T-cells, NKT-cells and gamma-delta cells (31). Furthermore, it consistently changed the microenvironment in several animal models (pancreatic and gastric) leading to a synergistic effect with checkpoint inhibitors (31). The prevention of metastases in mice treated with PAS (44) was due to the inhibition of epithelial mesenchymal transition, and this mechanism of action may help explain the long-term survivors previously observed in the clinical program. The decreased fibrosis observed with PAS therapy may help to explain the synergy previously found with gemcitabine. PAS vaccination in a precancerous KRAS murine model demonstrated that PAS not only decreases pancreatic fibrosis and alters the immune cell signature of the tumor microenvironment but that it also decreases proliferation and progression on precancerous PanIN lesions preventing pancreatic cancer (45). The data are compelling for PAS to return to the clinic with a much better understanding of how to use the product.

## Data Availability Statement

The raw data supporting the conclusions of this article will be made available by the authors, without undue reservation.

## Ethics Statement

The animal study was reviewed and approved by the Georgetown University IACUC.

## Author Contributions

Conception and design: JS, TP, LS, and AC. Acquisition of data: HC, JS, TP, KM, and WC. Analysis and interpretation of data: JS, HC, WC, KM, TP, LS, and AC. Writing, review and/or revision of manuscript: All authors. All authors contributed to the article and approved the submitted version.

## Funding

The study was funded in part by a grant from Cancer Advances, Inc., and its subsidiary Vaccicure, and NIH CA051008 to the Georgetown Lombardi Cancer Center Core facilities.

## Conflict of Interest

Authors TP, AC and LS are employees of Cancer Advances, Inc. and the company owns the patent rights to PAS.

The remaining authors declare that the research was conducted in the absence of any commercial or financial relationships that could be construed as a potential conflict of interest.

The authors declare that this study received funding from Cancer Advances, Inc. through a sponsored research agreement with Georgetown University. The funder had the following involvement with the study: Conception and design, Acquisition of data, Analysis and interpretation of data, and Writing, review and/ or revision of manuscript.

## Publisher’s Note

All claims expressed in this article are solely those of the authors and do not necessarily represent those of their affiliated organizations, or those of the publisher, the editors and the reviewers. Any product that may be evaluated in this article, or claim that may be made by its manufacturer, is not guaranteed or endorsed by the publisher.
